# Advancements in non-thermal technologies for enhanced extraction of functional triacylglycerols from microalgal biomass: A comprehensive review

**DOI:** 10.1016/j.fochx.2024.101694

**Published:** 2024-07-25

**Authors:** Harsh B. Jadhav, Pintu Choudhary, Nikhil D. Deshmukh, Dhananjay Kumar Singh, Moumita Das, Arpita Das, Nadiminti Chandana Sri Sai, Gayathri Muthusamy, Uday S. Annapure, Seema Ramniwas, Robert Mugabi, Gulzar Ahmad Nayik

**Affiliations:** a Department of Food Technology, Amity Institute of Biotechnology, Amity University, Jaipur, India; b Department of Food Technology, CBL Government Polytechnic Sector 13, HUDA, Bhiwani, Haryana 127021, India; c Department of Food Engineering and Technology, Sant Longowal Institute of Engineering and Technology, Sangrur, India; d Department of Food and Nutrition, Swami Vivekananda University, Barrackpore, Kolkata, India; e Department of Food and Nutrition, Brainware University, Kolkata, India; f Department of Dairy Sciences and Food Technology, Institute of Agricultural Sciences, Banaras Hindu University, Varanasi, Uttar Pradesh, India; g Department of Agricultural Microbiology, Tamil Nadu Agricultural University, Coimbatore, Tamil Nadu, India; h Department of Food Engineering and Technology, Institute of Chemical Technology, Matunga, Mumbai 400019, India; i University Centre for Research and Development, Chandigarh University Gharuan, Mohali, Punjab, India; j Department of Food Technology and Nutrition, Makerere University, Kampala, Uganda; k Department of Microbiology, Marwadi University, Rajkot, Gujarat 360003, India

**Keywords:** Microalgae, Extraction, Ultrasound, Ionic liquid, Supercritical CO_2_, Enzyme assisted, Functional triacylglycerols

## Abstract

Microalgae have emerged as a storehouse of biologically active components having numerous health benefits that can be used in the formulation of nutraceuticals, and functional foods, for human consumption. Among these biologically active components, functional triacylglycerols are increasingly attracting the attention of researchers owing to their beneficial characteristics. Microalgae are excellent sources of triacylglycerol containing omega-3 and omega-6 fatty acids and can be used by the vegan population as a replacement for fish oil. The functional triacylglycerols extracted using conventional processes have various drawbacks resulting in lower yield and inferior quality products. The non-thermal technologies are emerging as user-friendly and environment-friendly technologies that intensify the yield of final products and maintain the high purity of extracted products that can be used in food, cosmetic, pharmaceutical, and nutraceutical applications. The present review focuses on major non-thermal technologies that can probably be used for the extraction of high-quality functional triacylglycerols from microalgae.

## Highlights

• Microalgae are an excellent vegan source of functional triacylglycerols.
• Recent advances in the extraction of triacylglycerols from microalgal biomass are reviewed.
• Non-thermal extraction methods are potential replacement for the conventional extraction methods.
• Non-thermal extraction technologies are environment-friendly, economical, and give higher yields in a shorter time.

## 1 Introduction

The present market trend and the increase in demand from consumers for healthy food products are the driving forces for the use of microalgae for food production ingredients that are nutrition-rich, biologically active, and come from natural sources. The present-day consumer has become more prepense in choosing food products and is attracted more towards food products that contain natural and healthy food ingredients. The demand from consumers has turned food scientists and researchers to explore sources like microalgae for the extraction of functional ingredients that can be used in the formation of functional food products (Anusree et al., 2023; León-Vaz, León, Vigara, & Funk, 2023). The secondary metabolites formed by the microalgae are biologically active and apply to a variety of food and medicinal formulations to prevent lifestyle-related / non-communicable diseases (Raposo & De Morais, 2015; Sousa et al., 2023; Sousa, Pereira, Vicente, Dias, & Geada, 2023; Vaz, Moreira, de Morais, & Costa, 2016). Microalgae are the main components of the food chain as they are the primary producers, also they contain chlorophyll pigment which allows them to perform photosynthesis (Udayan, Pandey, Sharma, Sreekumar, & Kumar, 2021). Solar energy, nutrients, and carbon dioxide are utilized by microalgae to synthesize lipids, proteins, and carbohydrates. The cellular components of microalgae are utilized to manufacture biorefinery products with an extensive array of applications (Udayan et al., 2021). Microalgae include lipids that can be classified as either neutral or polar. The neutral lipids consist of polyunsaturated fatty acids, such as omega-3 and omega-6, while the polar lipids include phospholipids and glycolipids, which are typically found in the cell membranes of microalgae. (Mimouni, Couzinet-Mossion, Ulmann, & Wielgosz-Collin, 2018). The percentage of fat in microalgae is determined by several factors, including pH, temperature, light availability, and nutritional medium. Alpha-linolenic acid (ALA), eicosapentaenoic acid (EPA), and docosahexaenoic acid (DHA) are among the polyunsaturated fatty acids found in functional triacylglycerols, which are found in microalgae, notwithstanding their diversity. These functional triacylglycerols have been increasingly researched due to their ability to prevent non-communicable diseases and are among the essential components (essential fatty acids) needed for normal physiological functioning of the human body, as they are not synthesized in the human body, hence they form an important part of the human diet (Peltomaa, Johnson, & Taipale, 2018; Ward & Singh, 2005). A regular intake of these functional triacylglycerols in diet can help reduce the risk of cardiovascular diseases, reduce hyperlipidemia, and heart stroke, prevent atherosclerosis, and also help in the development of brain cells, maintaining good eyesight in infants. The functional triacylglycerols have proven to be nutrition-rich not only for adults but also for growing children (Lupette & Benning, 2020). The extraction of such functional triacylglycerols from the microalgae is equally important as the extraction method decides both the quality and quantity of the final product extracted.

The most common technique that is usually employed for the extraction of functional triacylglycerols from microalgae is solvent extraction. In this technique, the sample is repeatedly exposed to solvent, which ensures complete extraction of the product from microalgal biomass. Though this method is operator-friendly, simple, and cost-effective, still there are many challenges associated with this method the use of a large quantity of solvent, the extraction time is usually very high which has made researchers search for some environment-friendly technologies for extraction of functional triacylglycerols from microalgae (Aravind, Barik, Ragupathi, & Vignesh, 2020). The improvement and growth in technology have come up with innovative ways to overcome the challenges associated with conventional solvent extraction technology. The non-thermal technologies are emerging as a probable extraction tool that gives higher extraction yield in shorter extraction time with or without solvent. The feedstock in non-thermal technology is not exposed to higher temperatures, which maintains the quality of the final product (Table 1) (Xu, Zhao, & Su, 2021). For example, extraction of functional triacylglycerols from microalgae at higher temperatures may result in oxidative damage to the product, thus reducing the quality of the extracted product. A non-thermal process extracts at room temperature, preserving product quality throughout the process, using less solvent, producing a higher yield in a shorter amount of time, and is regarded as an environmentally and energy-efficient technology (Adam, Abert-Vian, Peltier, & Chemat, 2012; Araujo et al., 2013). Reviewing non-thermal technologies such as sonication, pulsed electric fields, high-pressure extraction, supercritical fluid, ionic liquid, etc. that are employed in the process of removing functional triacylglycerols from the microalgal matrix is the goal of this review. Additionally, the review contrasts these non-thermal technologies, provides details on the lipid and fatty acid composition of microalgae, investigates the possible use of functional triacylglycerols in the food industry, and ends with a closing statement that highlights future opportunities in the field covered in the review.

**Table 1:** An overview of non-thermal extraction technologies for extraction of functional triacylglycerol from microalgae showing merits and demerits.

Extraction Technology	Concept	Advantages	Disadvantages	References
High-Pressure Processing	Uses high pressure (100–600 MPa) to disrupt cell walls, enhancing the release of intracellular compounds	– Retains nutritional and functional properties of extracts – No heat degradation	– High operational costs – Limited scalability	Bueno et al., 2020
Supercritical Fluid Extraction	Uses supercritical carbon dioxide (above 31.1 °C and 7.38 MPa) as a solvent for lipid extraction	– High selectivity and purity of extracts – Environmentally friendly – No solvent residues	– High initial investment – Complex equipment requirements	Andrich, Nesti, Venturi, Zinnai and Fiorentini, 2005
Ultrasonic Assisted Extraction	Uses ultrasonic waves to create cavitation bubbles that disrupt cell walls and enhance mass transfer	– Short extraction times – Low solvent usage	– Limited to small-scale operations – Potential degradation of heat-sensitive compounds	Hui, Meng, & Kassim, 2023
Subcritical Water Extraction	Uses water at high pressure (subcritical state, 100–374 °C) to enhance the solubility and extraction of lipids	– Non-toxic and environmentally friendly solvent – High extraction efficiency	– High energy consumption – Potential hydrolysis of sensitive compounds	Ho, Kamal, Danquah, & Harun, 2018
High Hydrostatic Pressure	Applies high pressures (up to 600 MPa) uniformly to the biomass to enhance permeability and extraction of intracellular components	– Preserves functional and nutritional properties – Minimal heat degradation	– High cost of equipment – Limited scalability	Sousa, Carvalho, et al., 2023; Sousa, Pereira, et al., 2023
Pulsed Electric Field	Uses short bursts of high voltage electric fields to permeabilize cell membranes, enhancing the release of intracellular contents	– Low energy consumption – High extraction efficiency – Non-thermal	– Limited scalability – High initial costs	Joannes, Sipaut, Dayou, & Mansa, 2015

## 2 Microalgae

Microalgae are commonly known as seaweeds, which can be categorized as microalgae and macroalgae. Microalgae are photosynthetic microorganisms acting as miniature sunlight-driven cellular factories, which obtain energy with the aid of chlorophyll and other accessory pigments. Microalgae can grow under harsh environmental conditions are found in both aquatic and terrestrial and are very effective carbon dioxide fixers (Morales, Aflalo, & Bernard, 2021). Microalgae can also be found in salty water and effluents, avoiding fertilizer use, habitat degradation, and competition with other agricultural areas (Xue et al., 2018). Microalgae are primordial plants that are classified as Thallophytes. They are characterized by the absence of stalks, leaves, and roots, and their major photosynthetic pigment, chlorophyll. Additional examples of Thallophytes include slime molds, lichens, certain types of bryophytes, and fungi. The use of microalgae and fungus as biofuel sources has been extensively researched. Land plants, whose photosynthetic apparatus originated from microalgae, are thought to be the main contributors to the aquatic ecosystem. The biomass obtained from microalgae has been used for preparing food products, fuel, medicine, and other products since ancient times (Udayan et al., 2023). Rapid uplift in the Global human population has been alarming due to the increased demand for food, water, and many other energy sources. The need for excessive agricultural production was being forced by the increased demand for food, which is threatening the climatic and ecological factors. In this regard, there is a necessity to ensure reduced greenhouse gas emissions and an additional resource to meet the demands of the huge population. Approximately 30,000 distinct kinds of marine organism metabolites can potentially be found in the ocean, which makes up 70% of the surface of our planet. These metabolites have become a treasure trove of high-value compounds. In varied fields like food, medicine, materials, and other fields, these chemicals found their way through their application. About three-quarters of these bioactive chemicals derived from maritime environments are attributed to algae. As an autotrophic, photosynthetic organism, microalgae are rich in bioactive substances including lipids, proteins, and polysaccharides that are used in fuel, food, and medical industries, among other sectors. Because of their high unsaturated fatty acid (UFA) content, microalgae have been identified as an appropriate supplier of marine lipids and can be utilized in place of or as a substitute for fish-based oil. Microalgae lipids have significant amounts of eicosapentaenoic (EPA) and docosahexaenoic (DHA), which are crucial to immunological control, antibacterial activity, and inflammation prevention. These properties are also necessary for the production and use of microalgae lipids (Zhou et al., 2022). Wax, sterols, hydrocarbons, glycerolipids, and other lipid-like substances are produced by microalgae. With regard to microalgae lipid classes, glycerolipids are the most prominent and widely investigated. One, two, or three fatty acid (FA) groups are added to a glycerol backbone to delineate them (Morales et al., 2021). *Thraustochytrium*, *Schizochytrium*, *Aurantiochytrium*, and *Crypthecodinium cohnii* (*C. cohnii*) are examples of harmless microalgae that are rich in ω-3 fatty acids. DHA makes up about 60% of the fatty acids in *C. cohnii*. Microalgae's high PUFA content lowers production costs and boosts production efficiency (Li et al., 2019). One of the most promising dietary components for humans is polyunsaturated fatty acids, and consuming them in the right ratios (omega-3 to omega-6; 1:1 to 1:4) is vital for preventing chronic illnesses and non-communicable diseases like cardiovascular diseases, atherosclerosis, heart stroke, etc. (Katiyar & Arora, 2020). With its significant quantity of oil and unique fatty acid alignment, microalgae may be the most promising source of edible oils, with potential uses in food manufacturing.

### 2.1 Microalgal triglycerides

Microalgae is considered to be an excellent source of different functional triacylglycerols. The lipids in microalgae majorly comprise of two categories namely polar and neutral lipids. Phospholipids and glycolipids are polar lipids found in microalgae. In microalgae, the main energy requirement is fulfilled by neutral lipids (Lupette & Benning, 2020). Microalgal triacylglycerols (TAGs) are crucial raw materials to a large extent in the production of biofuels. In recent research, it has been reported that microalgal TAGs might serve as edible oil in the food industry and feed purposes and also might be utilized as a vehicle for lipophilic compounds such as carotenoids (Shen et al., 2016). As mentioned earlier microalgae biomass is known for producing large content of functional triacylglycerols including docosahexaenoic acid (DHA) and eicosapentaenoic acid (EPA). The fatty acid composition in functional triacylglycerols constituents varies greatly depending on the microalgae species. For example, it has been reported that TAGs in the algae *Chlamydomonas reinhardtii* comprise more than 50% of C18:1 and C16:0, whereas in the species *Arabidopsis thaliana*, major fatty acids found in TAGs are C16:0, C18:0 and C18:1. Another variety of microalgae *Isochrysis zhangjjangenesis* contain the functional triacylglycerols comprising C18:1 or C18:4 fatty acids (Shen et al., 2016). In the stress condition, a substantial quantity of TAGs can be produced among the microalgae like as *Chlorella vulgaris*, *Chromochloris* (syn. *Chlorella*) *zofingiensis*, *Chlorococcum littorale*, *Nannochloropsis oceanica*, *Neochloris oleoabundans* and *Scenedesmus obliquus*. The current trend of research explored various biotechnological aspects to induce the accumulation of functional triacylglycerols and other high-value components in microalgae for wider application in the sector of food, feed, and fuel markets.

Polyunsaturated fatty acids, which comprise essential fatty acids that must be consumed through diet, are vital for sustaining and enhancing human health. The microalgae have become a key source to meet this demand for functional triacylglycerols which can either be used in functional food or can be consumed in the form of tablets, capsules, etc. The lipid content in the microalgae varies with the conditions in which they are grown and is also governed by the type of microalgal species, roughly the lipid content varies between 1 and 85% (dry weight) in microalgae (Buono, Langellotti, Martello, Rinna, & Fogliano, 2014; Chacón-Lee & González-Mariño, 2010). Like the lipid content, the percentage of EPA, DHA, and ALA also varies in the microalgae and depends on the growing conditions and type of microalgal species (Gladyshev & Sushchik, 2019). The microalgae producing DHA (22C:6) can be categorized based on their ability to produce DHA. For example, the dinophytes can produce more than 40% DHA, the DHA content of euglenoids can go up to 60% contributed by the microalgae from genus *Schizochytrium* and *Aurantiochytrium,* and in haptophytes, the DHA content goes up to 30% (total fatty acids). Microalgal species like *Phaeodactylum tricornutum* and *Nannochloropsis sp* can produce up to 20% EPA and a small quantity of DHA (da Costa et al., 2017; Fan, Jiang, Faan, & Chen, 2007; Lee Chang et al., 2014; Mendes, Reis, Vasconcelos, Guerra, & Lopes Da Silva, 2009; Usup, Hamid, Chiet, Wah, & Ahmad, 2008). The EPA content of microalgal species *Nannochloropsis oculate* and Eustigmatophyte is approximately about 30% and 15% respectively (Yao, Gerde, Lee, Wang, & Harrata, 2015). Species like *T. lutea*, *P. lutheri, Skeletonema sp, and O. aurita* have higher percentages of omega-3 fatty acids. The saturated and monounsaturated fatty acids are also produced by microalgal species *Emiliania huxleyi, Synechococcus spp,* and *Nannochloropsis salina* (Morais et al., 2021)*.* Though the total lipid and the fatty acid content varies among the microalgal species, however, a change in the growth conditions during the cultivation of microalgae like variation in temperature, pH, nutrient culture, and light can result in increasing the functional triacylglycerol content in the microalgae. The decreases in the rate of cell productivity also enhance the lipid content in microalgal cells, which is usually observed when the microalgal cell undergoes a stressed situation. Hence, microalgae form an important source of functional triacylglycerol as their content can be managed depending on the growing conditions of the microalgae (Bellou et al., 2014).

## 3 Extraction technology

Functional triglycerides within microalgae possess significant bioactive components that are targeted for extraction for therapeutic purposes. Microalgal lipid extraction is an essential procedure for harnessing its bioactive components for various applications. The extraction process is intricate due to the unique characteristics of microalgae, including a thick-walled, rigid cell structure and a diverse array of lipid classes and triacyl glycerides, distinguishing it from conventional structures such as vegetable oils and foods (Breuer et al., 2013; Ryckebosch, Muylaert, & Foubert, 2012). The technology used in the extraction of microalgal triglycerides is broadly categorized into conventional and non-conventional methods. The conventional methods are the classical approaches used in lipid extraction, including Folch, Bligh-Dyer, and Soxhlet extraction. Bligh & Dyer and Folch, involve a methanol and chloroform combination for lipid release, but their scalability is questioned. These conventional methods operate in a simple manner and maintain a low budget, promoting their use. Alongside, it also imposes disadvantages like excess consumption of organic solvents and extraction time, posing a threat to environmental safety (Breuer et al., 2013; Ryckebosch et al., 2012). Additionally, they might not be ideal for the effective release of lipids from the strong microalgal cell wall. Effective lipid extraction requires breaking down the microalgal cell wall, which is the initial stage of the extraction process. (Ryckebosch, Bruneel, Termote-Verhalle, Muylaert, & Foubert, 2014) Different cell disruption methods paired with different solvents result in variations in lipid content, which may affect the makeup of fatty acids. (Breuer et al., 2013; D'oca et al., 2011; Florentino de Souza et al., 2014; Ryckebosch et al., 2012, Ryckebosch et al., 2014). The effectiveness of triglyceride extraction hinges on the chosen method, emphasizing the need for speed, efficiency, and economic viability (Chisti, 2007; Greenwell, Laurens, Shields, Lovitt, & Flynn, 2010). The yield of lipid content varies based on the extraction method chosen, influenced by fatty acid solubility and the solvent's ability to permeate the ruptured cell membrane. These techniques must be quick, scalable, and non-damaging to bioproducts (Brennan & Owende, 2010; Dong, Knoshaug, Pienkos, & Laurens, 2016; Mubarak, Shaija, & Suchithra, 2015; Pragya, Pandey, & Sahoo, 2013).

The choice of pre-treatment impacts the moisture content of the biomass, which might be either wet or dried (Chatsungnoen & Chisti, 2016; Halim, Gladman, Danquah, & Webley, 2011; Park, Park, Lee, & Yang, 2015; Taher, Al-Zuhair, Al-Marzouqi, Haik, & Farid, 2014). Triglycerides are extracted utilizing extraction solvents from the cell matrices. The subsequent steps involve separating lipids from cellular debris, isolating them from the solvent for extraction and residue water, and converting them to biodiesel through fractionation or transesterification based on desired lipid classes (Halim, Danquah, & Webley, 2012). Cell disruption generates various particle sizes of cellular debris, necessitating their removal through separation techniques like filtration and centrifugation. The miscible lipids in the solvent are separated via solid-phase absorption techniques, vacuum evaporation, or distillation. In lipid extraction technology, organic solvents or supercritical fluids are frequently employed. Cell disruption techniques are often used in conjunction with solvent-based processes. Lipid extraction involves the use of a variety of solvents, although depending on the target chemical's polarity, the use of solvents such as methanol, n-hexane, and chloroform can increase efficiency. (Balasubramanian, Allen, Kanitkar, & Boldor, 2011). However, these solvent-based techniques are energy-intensive and costly, requiring additional energy for solvent recovery (Iqbal & Theegala, 2013). Non-thermal methods, including pulsed electric field, sonication, enzyme-assisted, supercritical fluid extraction (SFE), ionic fluid, and high pressure, offer innovative alternatives overcoming the limitations of conventional approaches.

### 3.1 Conventional process

The Soxhlet extraction method, introduced in 1879, is a well-established technique for lipid extraction. It involves repeatedly contacting the sample with the extractant, enhancing the extraction yield. The Soxhlet extraction method involves three primary divisions: a round bottom flask that is constantly heated, a Soxhlet extractor for holding microalgae biomass, and a condenser for continuous cooling that provides a continuous replenishment of fresh solvents to the biomass, minimizing solvent consumption. This method has been utilized with various solvent systems, including chloroform, ethanol, hexane, methylene chloride, and methanol, to obtain lipids from microalgae like *chlorella*. Despite its advantages, after the Soxhlet extraction method, energy-intensive distillation is required to separate lipids from solvents (Neto et al., 2013). Soxhlet extraction (SE) is a technique that moves partially soluble components from a solid sample into a liquid phase (solvent) by using a Soxhlet extractor. Neutral lipids are extracted using nonpolar solvents like hexane. The solid sample is put inside the Soxhlet apparatus's main chamber in a filter paper thimble as part of the procedure. The solvent is then heated to reflux temperature, which enables it to enter the main chamber and extract the components from the sample that are less soluble. Using more polar solvents could increase the yield of microalgal extraction and make it easier to recover complex lipids and pigments (Baumgardt et al., 2016). Total lipid extracts produced with polar solvents are complex and contain metabolites in addition to lipids, therefore this is an important factor to examine. Crucial criteria for Soxhlet extraction include the choice of solvent, size of sample particles, and length of extraction (Sharif et al., 2014). The fundamental principle guiding the lipid extraction from microalgae utilizing solvents is rooted in the chemistry concept of ‘like dissolving like.’ This principle dictates that an ideal solvent should exhibit a high degree of specificity towards lipids, particularly triacylglycerols. Additionally, the solvent is obligated to possess sufficient volatility to facilitate low-energy distillation, ensuring efficient lipid separation from solvents. Both polar solvents such as ethanol, methanol, ethyl acetate, and acetone as well as non-polar solvents like benzene, diethyl ether, hexane, toluene, and chloroform are used in the extraction process. (Neto et al., 2013).

Among the various solvent systems employed, the chloroform/methanol (1/2 *v*/v) combination stands out as the most commonly operated system of organic solvents for lipid extraction from microalgal biomass and animal tissues. This solvent system is favored for its reduced extraction time and increased yield. Different solvent systems have been evaluated for their performance, such as dichloromethane/methanol, hexane/isopropanol chloroform/methanol, acetone/dichloromethane, and dichloromethane/ethanol. The selection of a solvent system significantly influences lipid yield, as demonstrated in studies involving microalgae species like *Botrycoccus braunii* and Pavlov (Cheng et al., 2011). Factors such as biomass drying, solvent system selection, and moisture content, including hexane: methanol and chloroform: methanol, have been investigated for their impact on intracellular lipid extraction from marine microalgae (Balasubramanian et al., 2011). One of the main disadvantages of the solvent extraction process for algal oil is the use of dangerous solvents like hexane and chloroform, which pose a serious risk to human health and the environment. (Aresta, Dibenedetto, Carone, Colonna, & Fragale, 2005). Work at the laboratory scale typically employs batch processes for oil extraction, where the solvent's lipid content reaches equilibrium, limiting additional lipid transfer in the batch process. While continuous organic solvent extraction methods overcome this limitation, they are expensive due to the large amount of organic solvent required. While Soxhlet extraction overcomes some of the drawbacks of traditional solvent-based methods, it still has limitations, including prolonged extraction times, lower yield (%), and substantial reagent consumption. Recent advancements in Soxhlet extraction, such as high-pressure Soxhlet extraction, aim to address these limitations and improve overall efficiency (Luque de Castro & Priego-Capote, 2010).

### 3.2 Non-thermal technology

#### 3.2.1 Pulsed electric field

**Fig. 1:**
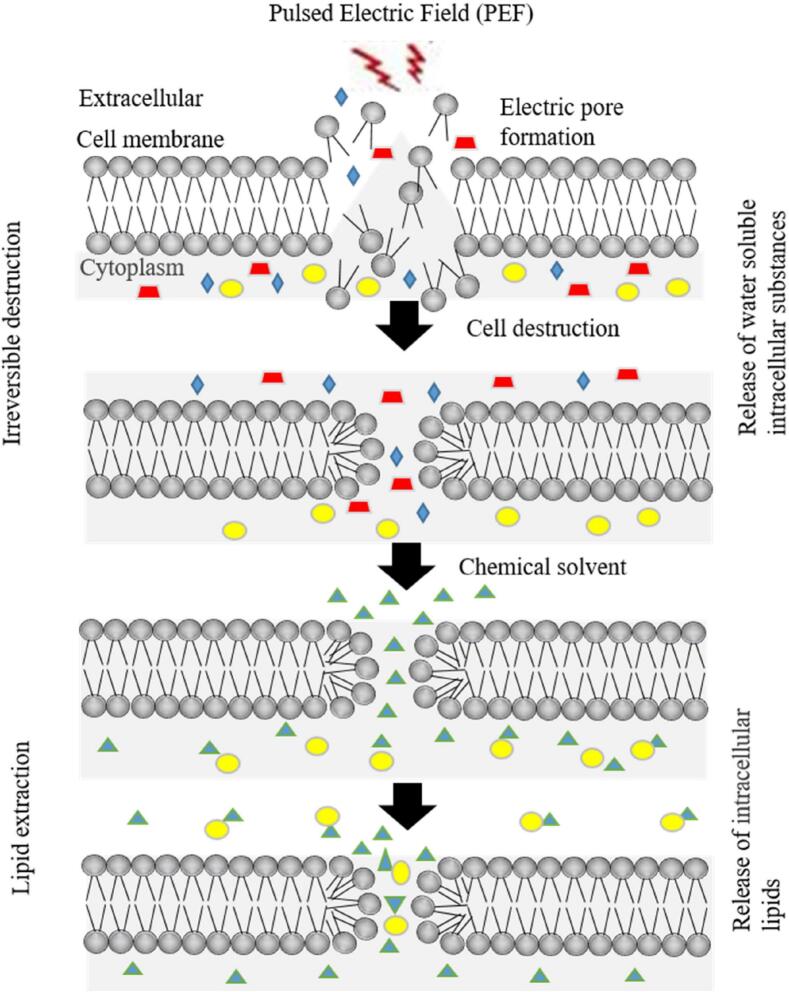
Extraction of triacylglycerols with application of pulse electric field. The symbol with red, blue, green, and yellow colors indicates protein, carbohydrates, triacylglycerols, and organic solvents respectively (Reproduced with permission from Elsevier, (Adapted from Han et al., (2019)). (For interpretation of the references to color in this figure legend, the reader is referred to the web version of this article.)

Pulsed Electric Field (PEF) technology emerged as a non-thermal processing method with diverse applications in the food industry, offering unique advantages for microalgae lipid extraction. PEF technology initially emerged for cell processing, but because of its adaptability to improve extraction procedures while maintaining food's nutritional content and sensory quality, it has become well-known in the food business. The fundamental idea behind PEF is called “electroporation,” in which a brief exposure to a high-voltage electric field ruptures the cell membrane and creates either transient or permanent pores (Fig. 1). The phenomenon of electroporation greatly enhances the permeability of microalgal cell walls, hence enabling the extraction of intracellular constituents, such as lipids.

The effectiveness of PEF-aided procedures in combination with ethanol-hexane solvent blends for lipid extraction from *Auxenochlorella protothecoides* and *Chlorella*, respectively (Silve et al., 2018; R. Zhang, Gu, Xu, & Fu, 2021). The outcomes indicated enhanced lipid recovery rates, reaching up to 90%, attributed to increased cell permeabilization and conductivity. The synergistic effects of PEF-assisted Bligh-Dyer extraction on *Chlorella pyrenoidosa* lipids (Han et al., 2019). The findings revealed a 12% higher lipid extraction rate compared to ultrasonic pre-treatment, emphasizing the role of PEF in promoting defects in the cell wall's surface, facilitating solvent-lipid interaction, and improving extraction efficiency. While PEF has shown promising results, limitations exist in its ability to extract larger molecular weight compounds. Studies by (Buchmann, Brändle, Haberkorn, Hiestand, & Mathys, 2019) and (Carullo et al., 2018) highlighted the lower extraction rates of proteins using PEF compared to high-pressure homogenization. Optimization strategies, including pre-enzymatic weakening of cell walls and multiple extraction cycles, have been proposed to overcome these limitations. PEF technology demonstrates superior energy efficiency compared to traditional methods such as bead milling. The low energy input of PEF makes it an environmentally friendly and economically feasible option for large-scale microalgal lipid extraction processes.

#### 3.2.2 Ultrasonic-assisted extraction

**Fig. 2:**
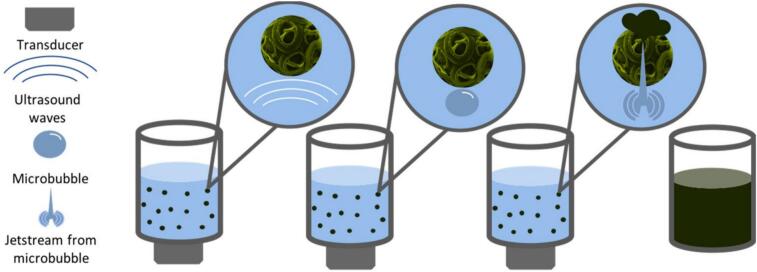
Extraction of triacylglycerol with application of ultrasound-assisted technology (Sosa-Hernández et al., 2019).

Ultrasound involves ultrasonic waves in a frequency range between 20 kHz to 10 MHz characterized as per the intensity, i.e., i. power ultrasound ranging from 20 to 100 kHz, having processing and extraction application, and ii. signal/diagnostic ultrasound which is having ranges from 100 kHz to 10 MHz, used for clinical diagnosis and quality assessment. Microalgae have a very thick cell wall that blocks the lipid release inside the cell. Conventional extraction processes like solvent extraction and mechanical pressing yield fewer lipids in comparison to the ultrasound-assisted extraction process (Mubarak et al., 2015). Acoustic cavitation is a physiochemical process that is included in the ultrasonic treatment for the extraction application. The expansion and contraction of pre-existing microbubbles in a system as a result of an ultrasonic field is known as acoustic cavitation (Fig. 2). (Ashokkumar, 2011). In the mechanism of ultrasound extraction, when the ultrasonic waves create pressure in the liquid media, bubble formation occurs. The formation of bubbles can occur in the ultrasonic extraction process by two methods i) gas present in the solid particle and ii) bubbles in the liquid media. The bubbles in the liquid media expand during each subsequent compression cycle in the ultrasonic extraction process from microalgae. Eventually, the bubbles reach a size where the ultrasonic energy is no longer enough to contain the vapors, and they collapse, causing mechanical and chemical effects that shatter the cell. (Liu, Liu, Cui, & Yuan, 2022).

**Fig. 3:**
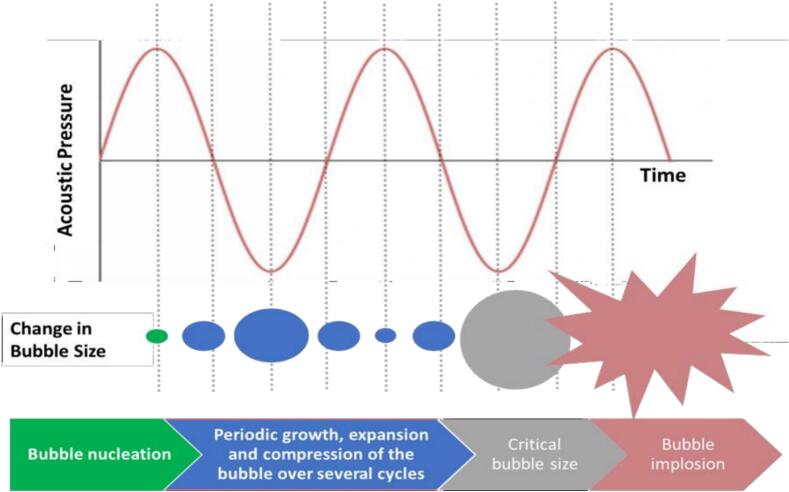
The mechanism of acoustic cavitation showing growth and bursting of the cavity bubble (Altay, Sadaghiani, Ilker Sevgen, Şişman, & Koşar, 2020).

The growth of these bubbles depends upon the frequency, ultrasound pressure, and the bubble ratio in the medium. The microbubble formation occurs during the introduction of ultrasonic waves. Growth and collapse occur after certain acoustic cycles, this is when the bubble bursts when it reaches a certain size (Fig. 3). High acoustic pressure, however, causes the bubbles to become unstable and burst. Damage to the solid surface is caused by macro-turbulence, which is created when bubbles in the liquid medium burst and grow. UAE (ultrasonic-assisted extraction) can improve the yield of the extracted compound, by the mechanism of collapsing bubbles, which damage the solid particle and release the compounds from the cells by damaging the cell wall. UAE enhances the mass and heat transfer between the solvent and matrix, and cavitation bubble generation and collapse facilitate solvent penetration into a matrix. This results in a ten-fold reduction in extraction time and a 50–500% increase in oil output from the microalgal biomass when UAE is used. The kind of solvent and the intensity of the ultrasonic waves can influence this. (Suali & Sarbatly, 2012).

Lipids from the microalgae cells were extracted by Adam et al. (2012) using an ultrasonic-assisted solvent-free extraction approach. The study was carried out using fresh *Nannochloropsis oculata* biomass, where the extraction was carried out at a low frequency (20 kHz) using a 1000 W ultrasonic processor, and power consumption and energy input were acquired. They concluded that the FAME's (Fatty acid methyl ester) amount in the solvent-free ultrasonic-assisted extraction was less in comparison to the conventional extraction process but the quality of fatty acid was preserved. Functional triglycerides can also be extracted from microalgal biomasses using ultrasonic extraction in conjunction with other methods. (Neto et al., 2013). The extraction process was used in the Widjaja, Chien, and Ju (2009) study on freshwater microalgae (*Chlorella vulgaris*). First, the material was ground up and then the mixture was extracted using a 2:1 ratio of chloroform to methanol. After 30 min of sonication, the sample's lipid extraction time was shortened by ultrasonication. On the yield, the ultrasonic treatment had no discernible impact, though.

#### 3.2.3 Enzyme-assisted extraction

Bioactive components in the microalgae matrix cannot be released as easily because of the thick and hard walls of microalgal cells. There is an immediate requirement for suitable and sustainable technologies to ensure efficient functional triacylglycerols extraction from microalgal biomass. Appropriate pre-treatment and extraction techniques can circumvent the need for additional steps in conventional methods and also maintain the bioactivity of lipophilic components. The recent innovative technologies mitigate the disadvantages of conventional processes to a certain extent but at the same, they require the installation of expensive equipment (Jeevan Kumar, Vijay Kumar, Dash, Scholz, & Banerjee, 2017). Enzyme-assisted extraction (EAE) is a well-established technique, that facilitates the release of intracellular bioactive components from microalgal matrix by cleaving the thick cell wall as shown in Fig. 1. The constituents of cell walls in the microalgal cells can hinder the functional triacylglycerols extraction efficiency. EAE technology is explored to substitute various traditional methods of functional triacylglycerols from microalgae. The specificity of the enzyme can aid in the selective breakdown of the compounds present in the rigid cell wall. Moreover, the method is mild, sustainable, and effectively reduces the consumption of energy (Y. Zhang et al., 2018). The study by Y. Zhang et al. (2022) found that employing the combination enzymes of cellulase, xylanase, and pectinase produced a 65.53% greater yield of functional triacylglycerols from microalgae *Scenedesmus* sp. than the untreated microalgae. It has been observed that the pace at which functional triacylglycerols are extracted from microalgae can be accelerated by combining enzymatic hydrolysis with other techniques. He et al. (2020) experimented with the effectiveness of three-phase partitioning (TPP) to extract functional triacylglycerols from marine *Nannochloropsis* strains in a combination with mixing four enzymes (cellulase, hemicellulase, papain, and pectinase). This integrated process is reported to be a promising technique ensuring the efficacy of superior functional triacylglycerols extraction in comparison with only enzymatic hydrolysis. The species *Chlamydomonas reinhardtii* is considered for accumulating functional triacylglycerols with a scope of application in food and pharmaceuticals. An advanced enzymatic treatment is proposed by Soto-Sierra, Wilken, and Dixon (2020) to improve the functional triacylglycerols extraction from microalgae *Chlamydomonas reinhardtii*. The study showed that aqueous enzymatic extraction can significantly improve the functional triacylglycerols and protein release from microalgal internal organelles. The experimental results of the study demonstrated that the inclusion of a primary (an enzyme specific for cell walls) and secondary enzymatic treatment (trypsin) increased the release of functional triacylglycerols by 73%. The EAE method has shown to be a successful method for improving the extraction of functional triacylglycerols from microalgae and for helping to separate different lipid fractions. To successfully rupture the microalgal cell wall and increase the extraction rate of functional triacylglycerols, the right enzymes must be chosen.

#### 3.2.4 Supercritical fluid extraction

**Fig. 4:**
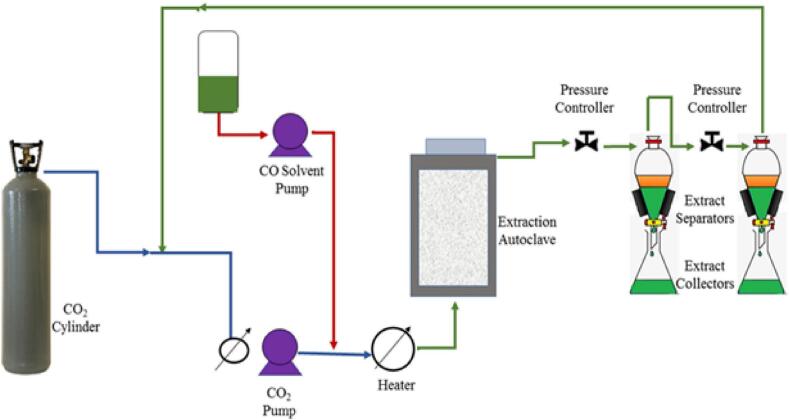
A representation of supercritical fluid extraction of functional triacylglycerols from the microalgal biomass (Maizatul, Radin, Mohamed, Ambati, & Ranga, 2021).

Among various innovative green technologies supercritical fluid extraction (SFE) method has gained attention of the researchers for its wide range of advantages. This process involves supercritical fluids using solvents for the extraction of various crucial components from a solid matrix. This method is a potent technique for the effective extraction of functional triacylglycerols from microalgal cells, as shown in Fig. 4. A supercritical state is referred to as a state when the temperature and pressure of a component are above the critical point. Components exhibit some unique characteristic features under a supercritical state, such as lower viscosity and density in comparison to liquids, diffusion properties like gas, and almost zero surface tension. The supercritical condition also enhances the solubility property of the supercritical fluids. These unique properties make the supercritical fluids better suitable for extracting bioactive compounds and also result in selective extraction with higher purity of the desired compounds (Tzima, Georgiopoulou, Louli, & Magoulas, 2023). Supercritical carbon dioxide, methanol, ammonia, and other substances are the most often utilized chemicals for SFE. Of them, supercritical carbon dioxide (CO_2_) is the most widely used by a variety of industries due to its affordability, environmental friendliness, and capacity to extract thermolabile bioactive materials, among other advantages. Moreover, the gaseous state of CO_2_ makes it easily separable from the extracts circumventing the requirement of solvent separation process (Zhou et al., 2022).

Wetterwald et al. (2023) investigation of the various supercritical fluid extraction technique settings revealed that the microalgae strain *Chlorella vulgaris* produced the best yield of functional triacylglycerols. In contrast to other SFE settings, the extraction yield at 40 °C and 200 bar was found to be 16.16 wt% extract/dry biomass. The simultaneous two-step extraction and fractionation of functional triacylglycerols from *Nannochloropsis* sp. microalgae were investigated by Jiménez Callejón et al. (2022). The combination of CO_2_ supercritical fluid extraction with a 10-wt% ethanol co-solvent produced a larger yield of functional triacylglycerols, which accounted for 81.7% of the biomass-neutral saponifiable lipids, according to their findings. The SFE approach is more effective than other methods at extracting functional triacylglycerols from microalgae like *Chlorella* sp. and *Phormidium* sp.*,* according to a recent study by Mathur et al. (2022). Comparing SFE to other conventional methods as the Soxhlet method (3.48%), a higher percentage of functional triacylglycerols (20.68%), including eicosapentaenoic acid, docosahexaenoic acid, α-linolenic acid, linoleic acid, and arachidonic acids, were recovered. Furthermore, Rodríguez-España et al. (2021) looked at how SFE affected the biomass of *Schizochytrium* algae's ability to extract functional triacylglycerols. Their findings showed that the optimum conditions for the highest yield were at 76.85 °C temperature and 46.52Mpa pressure, the use of ethanol as a co-solvent enhanced the efficacy of the method. The grinding pre-treatment had significantly impacted the efficacy of functional triacylglycerols extractability raising from 30% to 70% extraction efficiency. This pre-treatment also enhanced the DHA concentration in the extracted functional triacylglycerols and reduced the extraction time. Mouahid et al. (2020) investigated the processing conditions of supercritical CO_2_ extraction to obtain the highest yield of functional triacylglycerols from the microalgal *Nannochloropsis* species. They found that among the various parameters at 300 bar pressure and 323 K temperature, functional triacylglycerols yield was highest at 36.1 wt%.

#### 3.2.5 Ionic liquid

The process of extracting triglycerides from microalgae is considerably more challenging than that of removing triglycerides from oilseeds since microalgae are unicellular organisms with very robust cell walls that can be challenging to break. Separation is challenging because lipid-storing microalgae have low densities, and are present in suspensions. For triglyceride extraction, the organic solvents used in triglyceride extraction are typically highly concentrated and costly to recover. Studies have shown that 20–30% of the process's overall expenses are related to the removal of triglycerides from microalgae. Reducing the energy and production costs associated with triglyceride extraction, therefore, is a major area of attention for research. Most commonly, ethanol or methanol are employed as solvents in the chemical organic solvent extraction process. For triglyceride extraction, other common methods include Soxhlet extraction with hexane, extraction using dimethyl ether and isopropyl alcohol, and other subcritical organic solvent extraction using ethanol, hexane, or ethanol and supercritical carbon dioxide, as well as extraction using microwave systems and ultrasonic assistance. However, a lot of organic solvents are used in solvent extraction, and these solvents are typically poisonous, combustible, and have negative effects on the environment, public health, and security. Recovering the organic solvents from the extraction media is also expensive. Ionic liquids are green solvents better than green chemicals. These are being used for the separation of different materials from biomass. Ionic liquids are in a liquid state at 0–140 °C and contain larger asymmetric cations that are bonded with smaller inorganic or organic anions. The ionic liquid is considered to be the most desirable alternative for volatiles because of its non-volatile nature and heat resistance (Chen et al., 2015). The potential application of a rare type of hydrated phosphonium ionic liquid for the extraction of triglycerides from two different species of microalgae, *Chlorella vulgaris* and *Nannochloropsis oculata,* is as follows: These results have a lot of potential for creating a lipid extraction from microalgae process that is more effective. The often-used volatile organic solvents can be successfully substituted with the involatile ionic liquid. The standard triglyceride extraction process does not require a pre-treatment step to disrupt the microalgae cell wall due to the strong dissolving capacity of the ionic liquid. (Olkiewicz et al., 2015). An increased level of efficacy was shown when triglyceride extraction from *Chlorella sorokiniana*, *Nannochloropsis salina*, and *Galdieria sulphuraria* was done using ionic liquid 1-butyl-3-methylimidazolium hydrogen sulfate and microwave radiation in addition to traditional methods using organic solvents. Microwave radiation enhances the extraction process by 1990% in *C. sorokiniana*, 370% in *N. salina*, and 1170% in *G. sulphuraria* when compared to oil bath heating. Algal biomass can be effectively dissolved by HSO_4_, making it simple to extract the lipids. The process of extracting lipids from algae may be improved by the mineral acids (Pan et al., 2016). Ionic lipid extraction from microalgae could be more effective in comparison with other liquid extraction methods.

#### 3.2.6 High pressure

The process of pressurized liquid extraction, or PLE, has been extensively employed to extract bioactives from several strains of microalgae. When compared to traditional extraction methods, this extraction technique offers several benefits, such as enhanced mass transfer because of the specific extraction conditions used and faster extraction with smaller extraction solvent volumes. In fact, using liquid solvents at high temperatures causes their viscosity to decrease, which promotes the solvents' penetration into the matrix and, eventually, improves the extraction yield and kinetics. Ultra-high-pressure extraction (UHPE) is one type of pressured liquid extraction. In the latter instance, the pressure reaches values as high as 800 MPa, which is noticeably high. Plant cell structures may be weakened by the application of noticeably high pressures, obviating the necessity for an earlier cell disruption therapy (Gallego et al., 2021). Water is important in wet biomass extractions because it creates a layer at the cell wall that blocks many hydrophobic solvents from entering the cell. Extraction from wet microalgal biomass is challenging using hexane since it is a non-polar solvent and hydrophobic. For the freshwater chlorophyte microalgal consortium (Tarong polyculture), high temperatures and pressures lead to polarity changes in water, which render it miscible with hexane. So hexane was extracted under high pressure from the Tarong polyculture. With higher pressure and temperature at 50% sample dry biomass to water ratios, the greatest amount of fatty acids, mostly polyunsaturated membrane lipids, were found to be extractable (Islam, Brown, O'Hara, Kent, & Heimann, 2014). High pressure influences the effectiveness of lipid extraction along with solvents, which could be more effective in the conjugation of methods of extraction.

## 4 Concluding remark and future scope

Rapid globalization, urbanization, and deskbound lifestyles have been shown to increase the cases of non-communicable diseases across the globe. Consumers are demanding nutrition-rich food coming from natural sources and apart from fulfilling their hunger, the food should additionally have some health-friendly characteristics. Due to their abundance of physiologically active components, microalgae are considered one of the possible sources to satisfy the rising demand from consumers. The functional triacylglycerol derived from microalgal sources exhibits a greater proportion of polyunsaturated fatty acids in comparison to alternative sources such as fish oil. Because these fatty acids have qualities that suppress disease and are thought to be beneficial to human health, omega-3 and omega-6 fatty acids can be included in the creation of functional foods. Thus, food scientists are interested in researching the methods of microalgal functional triacylglycerol extraction. The increasing need for omega-3 and omega-6 fatty acids has compelled scientists to use greener, more advanced methods to extract functional triacylglycerols from microalgae. The non-thermal technologies have emerged as an energy and environment-efficient approach, that uses optimum temperature for extraction, gives higher yield (%) in shorter reaction time, and overcomes the disadvantages associated with the solvent extraction technology. The functional triacylglycerols can be used in various food applications like cookies, and pasta, or can be consumed in the form of pills, tablets, capsules, etc. Microalgae are a great source of omega-3 and omega-6 fatty acids and have the potential to replace fish oil, which the vegan community does not support. The functional triacylglycerol content of microalgae can vary depending on the growth conditions and when stressed, the lipid content increases. Therefore, it's critical to investigate potential non-thermal methods for obtaining functional triacylglycerol of high quality and quantity from microalgae. However, this field is still in its early stages of investigation and there is a lot to be explored in future research. Future studies should explore more microalgal species for their polyunsaturated fatty acid content and should develop strategies to increase their content by varying the appropriate growing conditions. In the future, the synergistic effect of two or more non-thermal approaches can be a beneficial step for getting higher yields and good quality products. This step will increase the probability of extracting functional triacylglycerols from microalgae, as today the entire biomass is used in the development of functional food products which negatively affects the sensory attributes and gives a dark green color to the product which has an impact on the consumer acceptance of the product. The extraction of functional triglycerides from the application of microalgae in the creation of functional foods is been expected to boost in the coming decades.

## CRediT authorship contribution statement

**Harsh B. Jadhav:** Investigation, Project administration, Supervision, Validation, Writing – original draft, Writing – review & editing. **Pintu Choudhary:** Conceptualization, Data curation, Resources, Software, Supervision, Writing – original draft, Writing – review & editing. **Nikhil D. Deshmukh:** Conceptualization, Formal analysis, Software, Supervision, Writing – original draft, Writing – review & editing. **Dhananjay Kumar Singh:** Formal analysis, Investigation, Resources, Software, Writing – original draft. **Moumita Das:** Conceptualization, Formal analysis, Methodology, Supervision, Validation, Writing – review & editing. **Arpita Das:** Conceptualization, Data curation, Resources, Software, Supervision, Writing – original draft, Writing – review & editing. **Nadiminti Chandana Sri Sai:** Data curation, Formal analysis, Methodology, Writing – original draft, Writing – review & editing. **Gayathri Muthusamy:** Conceptualization, Data curation, Formal analysis, Methodology, Resources, Writing – original draft. **Uday S. Annapure:** Conceptualization, Data curation, Formal analysis, Resources, Software, Writing – original draft, Writing – review & editing. **Seema Ramniwas:** Conceptualization, Data curation, Software, Supervision, Visualization, Writing – original draft, Writing – review & editing. **Robert Mugabi:** Formal analysis, Investigation, Resources, Software, Writing – original draft, Writing – review & editing. **Gulzar Ahmad Nayik:** Conceptualization, Data curation, Resources, Software, Writing – original draft, Writing – review & editing.

## Declaration of competing interest

The authors declare that they have no known competing financial interests or personal relationships that could have appeared to influence the work reported in this paper.

## Data Availability

Data will be made available on request.
